# Antimicrobial Resistance Profile of Group B *Streptococci* Colonization in a Sample Population of Pregnant Women from Romania

**DOI:** 10.3390/microorganisms12020414

**Published:** 2024-02-19

**Authors:** Aida Petca, Florica Șandru, Silvius Negoiță, Mihai Cristian Dumitrașcu, Daiana Anne-Marie Dimcea, Tiberiu Nedelcu, Claudia Mehedințu, Marinela Magdalena Filipov, Răzvan-Cosmin Petca

**Affiliations:** 1 Department of Obstetrics and Gynecology, “Carol Davila” University of Medicine and Pharmacy, 8 Eroii Sanitari Blvd., 050474 Bucharest, Romania; aida.petca@umfcd.ro (A.P.); mihai.dumitrascu@umfcd.ro (M.C.D.); daiana.dimcea@yahoo.com (D.A.-M.D.); claudia.mehedintu@umfcd.ro (C.M.); 2 Department of Obstetrics and Gynecology, Elias University Emergency Hospital, 17 Marasti Blvd., 011461 Bucharest, Romania; 3 Department of Dermatology, “Carol Davila” University of Medicine and Pharmacy, 050474 Bucharest, Romania; 4 Department of Dermatology, Elias University Emergency Hospital, 17 Marasti Blvd., 011461 Bucharest, Romania; 5 Department of Anesthesiology and Critical Care, “Carol Davila” University of Medicine and Pharmacy, 050474 Bucharest, Romania; 6 Department of Anesthesiology and Critical Care, Elias University Emergency Hospital, 17 Marasti Blvd., 011461 Bucharest, Romania; 7 Department of Obstetrics and Gynecology, University Emergency Hospital, 050098 Bucharest, Romania; 8 Faculty of Medicine, “Carol Davila” University of Medicine and Pharmacy, 8 Eroii Sanitari Blvd., 050474 Bucharest, Romania; tiberiu.nedelcu2022@umfcd.ro; 9 Department of Obstetrics and Gynecology, Filantropia Clinical Hospital, 011171 Bucharest, Romania; 10 Department of Laboratory Medicine, Elias University Emergency Hospital, 17 Marasti Blvd., 011461 Bucharest, Romania; marilenafrunzoi@yahoo.com; 11 Department of Urology, “Carol Davila” University of Medicine and Pharmacy, 8 Eroii Sanitari Blvd., 050474 Bucharest, Romania; razvan.petca@umfcd.ro; 12 Department of Urology, “Prof. Dr. Th. Burghele” Clinical Hospital, 20 Panduri Str., 050659 Bucharest, Romania

**Keywords:** group B *Streptococcus*, antimicrobial resistance, pregnant women, *Streptococcus agalactiae*

## Abstract

Group B *Streptococcus* (GBS) represents one of the leading causes of life-threatening invasive disease in pregnant women and neonates. Rates of GBS colonization vary by region, but studies on maternal GBS status are limited in Romania. This study aims to identify the prevalence of colonization with GBS and whether the obstetrical characteristics are statistically associated with the study group’s antimicrobial susceptibility patterns of tested GBS strains. This observational study was conducted between 1 May and 31 December 2021 at The Department of Obstetrics and Gynecology at Elias University Emergency Hospital (EUEH) in Bucharest, Romania. A total of 152 samples were positive for GBS and included in the study according to the inclusion criteria. As a result, the prevalence of colonized patients with GBS was 17.3%. GBS isolated in this population had the highest resistance to erythromycin (*n* = 38; 25%), followed by clindamycin (*n* = 36; 23.7%). Regarding the susceptibility patterns of tested strains to penicillin, the 152 susceptible strains had MIC breakpoints less than 0.06 μg/μL. The susceptibility patterns of tested strains to linezolid indicated three resistant strains with low levels of resistance (MICs ranging between 2 and 3 μg/μL). Multidrug resistance (at least three antibiotic classes) was not observed. In conclusion, although GBS naturally displays sensitivity to penicillin, the exact bacterial susceptibility testing should be performed in all cases where second-line therapy is taken into consideration for treatment. We acknowledge the need for future actions to limit multidrug-resistant bacteria.

## 1. Introduction

*Streptococcus agalactiae* is a group B *Streptococcus*. The term ‘group B *Streptococcus*’ or GBS belongs to the group B pyogenic streptococci. It is the only *Streptococcus* species harboring the Lancefield group B cell wall-specific polysaccharide antigen common to all GBS strains [1].

GBS is a Gram-positive bacteria that naturally colonizes the gastrointestinal and rectovaginal tracts in 18% of women globally (95% CI, 17–19%) [2]. Risk factors for GBS colonization and invasive disease were demonstrated in a study, where Black or African race (aRR 1.48, 95% CI 1.41–1.54) and preexisting diabetes (aRR 1.12, 95% CI 1.01–1.23) were independently associated with a risk of colonization [3].

GBS is the most common cause of neonatal mortality and morbidity in the United States [4]. Infections in newborns occurring in the first seven days of life are designated as early-onset disease (EOD), while late-onset disease (LOD) infections occur in infants aged more than one week [4]. In comparison to GBS-EOD prevention, there are no strategies to prevent GBS infection among infants with illness onset between days 7–89 of life [5].

In countries with no streptococcal prophylaxis policy in pregnant women, it has been demonstrated that 1.1% (95% CI, 6–1.5%) of newborns from mothers screened positive for genital GBS develop symptoms. In contrast, in countries where antibiotic prophylaxis is applied, the risk drops to 0.03% (95% CI, 0–0.7%) [2].

GBS uses virulence factors encoded by different genes to invade, replicate, and persist in a host. Among these, the most identified virulence factors were identified: a bacterial capsule that includes antibodies specific to each serotype, the surface of Rib proteins, and the alpha and beta antigens of the C protein (coded by *rib*, *bca*, and *bac* genes) [6,7]. Antimicrobial resistance genes are found in the environment and give GBS the ability to resist the effect of antibiotic medication [8]. The increase in antibiotic resistance in the treatment of GBS generates concern in the case of administration in severe forms of infection [9,10].

Intravenous penicillin G is the first-choice antibiotic in intrapartum GBS infection prophylaxis [11]. In the absence of penicillin G, ampicillin is the therapeutic alternative; the loading dose of ampicillin is 2 g intravenously, followed by a maintenance dose of 1 g intravenously every 4 h until the delivery moment [11]. In most cases, GBS is susceptible to penicillin and ampicillin, but recent studies demonstrate the emergence of strains with low or intermediate susceptibility to both penicillin and ampicillin [12,13,14].

In the case of patients with a history of penicillin allergy but a low risk of developing anaphylaxis, the recommended antibiotic is cefazolin instead of clindamycin or erythromycin due to the increase in bacterial resistance [4,11]. Resistance to second-line therapy antibiotics, including lincosamides, of which clindamycin is part, or erythromycin, a macrolide, has increased over the past 15 years [15,16]. Resistance is determined by three genes: *ermB*, *ermA* (subclass *ermTR*), and *mefA/E*. Mechanisms that confer resistance to lincosamides and macrolides include target site modification by methylation of the 23SrRNA binding site. This mechanism is mediated by *ermB* and *ermTR* genes, which confer cross-resistance to all MLSB antibiotics [17]. The *mefA* și *mefE* genes encode 14- and 15-member macrolide efflux pumps and produce the macrolide-resistance phenotype (M phenotype) [18]. 

The high rate of erythromycin and clindamycin resistance in GBS strongly supports the current recommendation that antibiotic susceptibility testing should be performed if erythromycin or clindamycin therapy is needed to prevent neonatal GBS infection [19].

The Romanian Society of Obstetrics and Gynecology recommends GBS prophylaxis depending on the patient’s risk factors or in case of ruptured membranes after more than 12 h. However, in Romania, some obstetricians still recommend a therapeutic scheme consisting of two or more antibiotics in case of positive screening, contributing to the increase in bacterial resistance [20].

There are no data concerning the bacterial resistance of GBS and the prevalence of colonization in pregnant women from Romania. Therefore, the aim of this study is to identify the prevalence of colonization with GBS and to identify if obstetrical characteristics are statistically associated with the antimicrobial susceptibility patterns of tested GBS strains in the study group admitted to the Obstetrics Department from a hospital unit belonging to the National Health System from Bucharest, Romania.

## 2. Materials and Methods

### 2.1. Study Design, Scope, and Period

This observational study was conducted between 1 May and 31 December 2021 at The Department of Obstetrics and Gynecology at Elias University Emergency Hospital (EUEH) in Bucharest, Romania. It is a significant healthcare facility in Bucharest that supplies a total capacity of 75 beds in the Obstetrics and Gynecology department out of 1100 total size. It provides specialized, preventive, curative, emergency medical assistance and care in case of pregnancy and maternity, as well as for newborns, and carries out research and education programs. 

The inclusion criteria in the studied group were as follows: pregnant patients with a minimum age of 18 years old, positive for GBS vaginal screening at the moment of admission, third-trimester pregnancy, single or multiple pregnancies, intact or ruptured membranes, and the lack of use any antibiotic in the last three weeks prior to hospitalization. Informed consent was obtained from each patient. 

The exclusion criteria were pregnant patients with age less than 18 years old, a positive GBS vaginal screening culture during the current pregnancy or GBS bacteriuria during any trimester of the current pregnancy, pregnant patients who had taken antibiotics in the last three weeks prior to hospitalization, and pregnant patients who refused to consent for the study. 

The study was approved by the Ethics Committee of Elias University Emergency Hospital Bucharest (registration number 2517/7 April 2020).

### 2.2. Data Collection

Patient information was prospectively obtained. The following data were collected: (1) demographic data, such as maternal age; (2) clinical data, such as gestational age, parity, gravidity, number of fetuses in present pregnancy, outcomes of the previous and current delivery, premature rupture of membranes, gestational or prepregnancy diabetes, and known antibiotic allergies, and (3) laboratory data regarding the antibiotic classes used for the treatment of GBS in pregnancy, such as minimum inhibitory concentration (MIC90, MIC50) for benzylpenicillin, erythromycin, clindamycin, and linezolid. Minimum inhibitory concentration (MIC) is a tool used to determine the resistance to a specific antibiotic and guide the therapeutic process. The determination of the MIC is used to obtain an accurate result of the most effective antibiotic classes to treat GBS infection. 

### 2.3. Specimen Collection, Transport, and Processing

Vaginal samples were collected from pregnant patients before vaginal examination using a sterile cotton swab by qualified medical staff following universal precautions. Firstly, the swab was inserted approximately 2 cm intravaginally without the use of a speculum, and later, the samples were inserted into Amies transport medium (which maintains the temperature at 37 °C) and transferred to the microbiology laboratory from the hospital during the first 2 h after collection.

For the cultivation step, a selective enrichment medium for GBS was chosen (Todd Hewitt broth, NutriSelect^®^ Plus, Merck KGaA, Darmstadt, Germany), from which passages were made at 18 h on Columbia Agar + Sheep Blood Plus Agar (incubation in the atmosphere with 5–10% CO_2_, 16–24 h, 35–37 gr C) and on Brilliance GBS Agar (incubation in an aerobic atmosphere, 16–24 h, 35–37 gr C).

A positive screening is correlated with the presence of pink colonies developed on the Brilliance GBS Agar medium. For confirmation, at the same time, hemolytic β colonies isolated on Columbia agar + Sheep Blood were subjected to agglutination with sera specific for group identification (Thermo Scientific™, Brilliance™ GBS Agar, Dardilly, France).

### 2.4. Antimicrobial Susceptibility Determination

Antimicrobial resistance of GBS was performed according to the recommendations of the European Committee on Antimicrobial Susceptibility Testing (EUCAST) standard (2020 version) [21,22].

Two testing methods were used. The automated system Vitek2C (BioMerieux, Marcy l’Etoile, France) was used for all samples using the AST-ST03 kit. When needed, samples were further retested for susceptibility to four antibiotics used in the treatment of GBS infection in pregnancy through the disk-diffusion method. The disk-diffusion method used Mueller Hinton Agar with 5% defibrinated horse blood and 20 mg/L β-NAD with 1 unit of penicillin, 15 µg of erythromycin, 2 µg of clindamycin, and 10 µg of linezolid (Oxoid Ltd., Cheshire, UK) [23,24]. 

Reading of the results was practiced manually. Growth appears as turbidity or a deposit of cells at the bottom of the well. The appearance of growth differs depending on the antimicrobial agent tested [25].

MIC was determined based on the lowest concentration of each antimicrobial agent that causes a complete inhibition of bacterial growth [26].

Broth microdilution (BMD) endpoints are specific values of some parameters, such as MIC or inhibition zones, based on which bacteria can be classified into clinical categories: susceptible (sensitive), susceptible but increased exposure (intermediate), or resistant [27].

### 2.5. Statistical Analysis

IBM SPSS Statistics for Windows software, Version 29.0, was used for the statistical processing of the study data (30-day trial) (IBM Corp, Armonk, NY, USA).

The association between variables was analyzed using cross-tabulation and the χ^2^ (chi-square) test. If the chi-square test results were sufficiently altered so that they could not be considered, Fisher’s exact test was used.

The independent samples *t* test was used to compare means according to dichotomous variables in the study.

We used the ANOVA test to compare three or more group means where the participants are the same in each group.

A statistical significance coefficient value of *p* < 0.05 was considered significant.

## 3. Results

### 3.1. Demographic Characteristics of the Included Participants

The total number of patients who gave birth in the mentioned period was 878. A total of 152 samples were positive for GBS and included in the study according to the inclusion criteria. The prevalence of colonized patients with GBS was 17.3%. The calculated mean age for the studied group was 30.9 ± 7 years.

Most of the participants included in the study who were positive for GBS screening were 30–39 years (44.8%) and 20–29 years (44%). The maximum age of positive screening for GBS was 44 years (Table 1).

### 3.2. Obstetrical Characteristics of the Study Group and Antimicrobial Susceptibility Patterns of the Tested Strains

The presence of GBS in participants included in the study with gestational age < 35 weeks was 4.6%, and in patients with gestational age ≥ 36 weeks, it was 95.4%. The mean gestational age of participants was 39 weeks.

A little over half of the study group were primigravida participants (*n* = 77; 50.7%), while the rest were multigravida (*n* = 75; 49.3%). We compared primigravida with multigravida to see if there is a specific pattern of antibiotic susceptibility. Different proportions of the MIC of benzylpenicillin were not identified in these two groups (primigravida and multigravida, respectively). Therefore, we can state that there was no statistically significant association (χ^2^ = 6.072; *p* = 0.194) between gravidity and susceptibility pattern to benzylpenicillin. Furthermore, there is no statistically significant association between gravidity and susceptibility patterns for erythromycin (χ^2^ = 0.907; *p* = 0.635), for clindamycin (χ^2^ = 1.052; *p* = 0.591), or for linezolid (χ^2^ = 0.367; *p* = 0.544).

In our study group, there was not a statistically significant association between preterm labor among participants (<37 weeks of gestation) or a susceptibility pattern for benzylpenicillin (χ^2^ = 0.940; *p* = 0.919), erythromycin (χ^2^ = 2.061; *p* = 0.357), or linezolid (χ^2^ = 0.193; *p* = 0.661). However, in the group of participants who gave birth at term, a higher MIC (>0.5 μg/μL) was found for clindamycin compared to the preterm labor group. For participants with preterm labor, for the majority (88.9%), MIC for clindamycin was ≤0.25 μg/μL. We can state that preterm birth is significantly (*p* < 0.001) associated with MICs below 0.25 μg/μL.

Because of the reduced number of participants with multiple pregnancies, a history of intrauterine fetal death, or a history of antibiotic allergies, we were not able to identify a statistically significant result for any of the studied antibiotics. 

The prevalence of GBS infection in participants with a history of penicillin allergy was 1.3% (*n* = 2) (Table 2).

### 3.3. Antimicrobial Susceptibility Determination in Studied Participants

Antimicrobial susceptibility patterns of the selected 152 GBS-positive samples from the examined patients, expressed as MICs (μg/μL), and their absolute and relative frequencies are evaluated in Table 3.

The antimicrobial susceptibility patterns for penicillin, erythromycin, clindamycin, and linezolid were tested in the entire study group. GBS isolated in this population had the highest resistance to erythromycin (*n* = 38; 25%), followed by clindamycin (*n* = 36; 23.7%). The tested strains resistant to erythromycin showed a very high level of resistance with MICs ≥8 μg/μL in comparison to resistant strains to clindamycin that showed a less resistant pattern with MICs ranging between 1 and 3 μg/μL. A total of 33 out of 36 strains resistant to clindamycin were also resistant to erythromycin.

Regarding the susceptibility patterns of tested strains to penicillin, the 142 susceptible strains had MIC breakpoints less than 0.06 μg/μL. Five strains (3.3%) were resistant to penicillin (>0.125 μg/μL), and another five showed intermediate resistance to penicillin (3.3%) as a result of the first automatic testing. The ten strains that initially showed resistance and intermediate resistance to penicillin were retested through the two mentioned methods (automated system Vitek2C using the AST-ST03 kit, respectively disk-diffusion method), obtaining CMI values ≤0.06 μg/μL at the second test, which led to the strains being classified as susceptible. In conclusion, no strains resistant to penicillin were recorded.

The susceptibility patterns of tested strains to linezolid indicated three resistant strains with low levels of resistance (MICs ranging between 2 and 3 μg/μL). Only one of these strains was resistant to erythromycin as well.

Multidrug resistance (at least three antibiotic classes) was not observed. Extensively, drug resistance to all antibiotic classes was not observed. Resistance to two classes of antibiotics was found in 21% of cases (*n* = 32); the most frequent association of resistance was between erythromycin and clindamycin in 96.9% of two drug resistance cases (*n* = 31).

## 4. Discussions

GBS is an important cause of infection in pregnant women and newborns in many countries, but there are only a few valid data in Eastern Europe, especially in Romania.

Because of the potential risk of mother-to-newborn infection, screening for GBS in pregnancy is necessary, and these data are essential for interventions to prevent further infection. The rate of GBS colonization differs globally, depending on religion, ethnicity, geographic area, socioeconomic status, sample collection and storage methods, laboratory techniques, and maternal and gestational age [28].

The main early-onset route of neonatal infection is vertical transmission from colonized mothers during vaginal passage in natural birth [29]. Most newborns from colonized mothers do not develop symptoms or signs of early infection. In the absence of prophylactic measures, the early onset of neonatal infection occurs in up to 2% of cases in mothers colonized with GBS [30]. Additional risk factors for preterm infection include labor before 37 weeks of gestation, maternal febrile syndrome during labor (greater than 100.4 °F or 36 °C), and premature rupture of membranes (greater than 18 h) [4]. 

Studies demonstrated a less common horizontal transmission of infection from the mothers or community contacts. The cause of late-onset GBS infection presents a fecal–oral route of transmission from exogenous sources, such as breastfeeding or limited areas of colonization, but the results are uncertain [31]. Late-onset GBS infection occurs between 7 and 89 days of life (mean at day 37) and has a similar presentation to early onset [32].

Sample collection to determine GBS should be performed in every pregnant woman, as colonization may be temporary [4]. Screening has been shown to be a more practical approach for preventing early-onset neonatal disease caused by GBS than identifying maternal risk factors [33]. Identifying patients who require intrapartum prophylaxis is an important aspect. Thus, the Centers for Disease Control and Prevention (CDC) recommends screening methods through rectovaginal sample collection. It is recommended that cultures for GBS should be obtained between 35 and 37 weeks of gestation [4,11]. In the case of high-risk premature birth patients, the culture for determination of GBS should be performed before 35 weeks of pregnancy [4]. The rapid antigen detection method is not recommended to be performed instead of rectovaginal sample collection, as it has low sensitivity and specificity, but nevertheless, it can be recommended intrapartum in patients with undispensed pregnancy to prevent unnecessary administration of antibiotics [34,35].

Urinary tract infection with GBS is another form of colonization of the genital tract. In this sense, all pregnant patients should be evaluated for the detection of asymptomatic bacteriuria in pregnancy, and those with positive screening should receive intrapartum prophylaxis [4]. 

A review conducted by Russell N. et al. in 2017 included 82 articles (26%) that described testing for GBS colonization at the moment of delivery and 94 articles (30%) that described tested samples from pregnant women < 35 weeks of gestation. The prevalence of maternal GBS colonization in studies that reported samples from pregnant women before 35 weeks of pregnancy was 17% (95% CI, 15–18%), then 15% (95% CI, 13–16%) in those sampled after 35 weeks and 14% (95% CI, 13–16%) at the moment of delivery [2]. Another systematic review conducted by Abbasalizadeh F. et al. included 245 full texts that reported the prevalence of GBS colonization in pregnant women from all the studied countries was 15.5% (95% CI, 14.2–17%) [36]. Similar results were obtained in the present study, where the prevalence of colonized pregnant women included in the study was 17.3%.

Penicillin is the treatment of choice in GBS infections [37]. However, in vitro studies have indicated that this antibiotic’s rate of GBS eradication is relatively low compared to other streptococcal pathogens. This has led some authors to recommend dual therapy associated with gentamicin for severe forms of infection. This approach requires that the pathogen agent be susceptible to penicillin and aminoglycoside antibiotics [38]. Our study confirmed the natural sensitivity of GBS for penicillin, and all samples that initially were tested resistant proved sensible upon the standard second retest. Similar results regarding penicillin resistance were also identified in the survey conducted by Quiroga et al. [37]. As in this last-mentioned study, penicillin or ampicillin remains the antibiotic of choice for intrapartum prophylaxis of GBS infection. The in vitro results demonstrated that penicillin (MIC ≤ 0.125 μg/μL in 96.7% cases) is the antibiotic of choice in the studied population in Romania, the results being similar to those of another study from Belgium [39]. Although cases of GBS with reduced susceptibility to penicillin have begun to appear worldwide, intravenous penicillin remains the first-line therapeutic agent [40]. The macrolide and lincosamide classes of antibiotics share similar binding loci, and this is the main reason that resistance to one of these is usually transmitted to the other [21]. Thus, GBS resistance to clindamycin but not erythromycin (L phenotype) is rare [19]. This is demonstrated in a study conducted on pregnant patients in Vietnam, demonstrating that the L phenotype rate was 2.2%, higher than in a previous study (0.31%) performed in the USA [41,42].

In the case of penicillin-allergic patients, erythromycin and clindamycin are therapeutic alternatives used in intrapartum prophylaxis. Clindamycin is the first-line therapeutic agent in this case because of the lower resistance rate compared to erythromycin. In addition, clindamycin crosses the placental barrier more easily and causes higher concentrations of the antibiotic in the fetus [39]. However, it should be noted that 11% of streptococcal strains show cMLSB phenotype, resulting in increased resistance to erythromycin and clindamycin (MIC > 0.5 μg/μL). This resistance to erythromycin and clindamycin is caused by two main mechanisms in *Streptococcus* spp.: target site methylation of the 23S ribosomal component of the large (50S) ribosomal subunit and an active efflux pump. The methylase can be expressed constitutively or can be induced. This mechanism causes the pathogen to become resistant to most macrolides, lincosamides, and streptogramic B compounds (MLSb phenotype) [39,43]. In our study, 25% of GBS strains were resistant to erythromycin, and 23.7% were resistant to clindamycin. Compared to this, a study conducted in Vietnam demonstrated much higher percentages of resistance to these two antibiotics (76.23% resistance to erythromycin and 58.21% resistance to clindamycin) [42]. A study conducted in Korea occupies an intermediate position in terms of resistance to these two antibiotics (16% resistance to clindamycin and 28.8% resistance to erythromycin) [44]. This suggests that resistance to erythromycin and clarithromycin varies by geographic area. 

Noteworthy are data regarding the number of erythromycin- and clindamycin-resistant isolates, which indicate high rates (25% resistance to erythromycin and 23.7% resistance to clindamycin) of resistance to the most largely used antibiotics in cases of penicillin allergy. These results confirm the increasing incidence of macrolide and lincosamide resistance among GBS strains worldwide, suggesting that the use of these antibiotics should be opportunely preceded by routine susceptibility testing as we proceeded in this study.

A newer antibiotic was also included in the study. Linezolid is an oxazolidinone with good activity against Gram-positive organisms, such as GBS [45]. According to studies, linezolid has proved to be highly active in vitro against all of the GBS, and this is why it is used as an alternative therapeutical agent [45,46,47]. In the current study, sensitivity to linezolid was 98% (MIC ≤ 2 μg/μL in 149 strains). Similar results were also demonstrated in the study conducted by Haimbodi et al. in Namibia (100% of the evaluated strains were sensitive to linezolid), with MICs between 1 μg/μL and 2 μg/μL [48].

Multidrug resistance (at least three antibiotic classes) was not observed in our study. These results indicate a lower multidrug resistance compared to similar studies conducted in other countries on different ethnicities from Africa, such as Ethiopia (13.79%), and in countries from Asia, such as South Korea (60.66%) [42,49]. 

In many laboratories, the susceptibilities of GBS are not tested since isolates of GBS, as reconfirmed in the present study, are uniformly susceptible to penicillin in vitro. High in vivo treatment failure rates have led to the use of other antibiotics, such as erythromycin or clindamycin. The levels of antibiotic tolerance exhibited by GBS isolates in this study argue in favor of routine susceptibility testing of clinical isolates. As an update about GBS antibiotic susceptibility patterns in an era of widespread antibiotic use during pregnancy, we studied the susceptibilities of 152 GBS isolates to penicillin, clindamycin, erythromycin, and linezolid. Patterns of susceptibility to erythromycin and clindamycin revealed increasing resistance or intermediate susceptibility at higher rates than have been previously reported [50].

GBS resistance to various antibiotic classes in this study is concerning and indicates that alternative therapeutic strategies are needed sooner. Antibiotic resistance rate was the highest for erythromycin (25%), followed by clindamycin (23.7%), linezolid (2%) and penicillin (0%). Antibiotic resistance in our study is largely similar to that at a global level [39,48]. Monitoring the levels of antibiotic resistance is therefore important. The resistance of at least two antibiotic classes is alarming and justifies the need for further investigations.

An emerging therapeutic alternative to prevent neonatal and maternal disease caused by GBS is the vaccination of pregnant patients in the third trimester [51]. There are several candidate vaccines to prevent disease, including a variant of vaccine that acts predominantly against Ia, Ib, and III serotypes, which is currently under development [52]. Recent studies showed that the development of a vaccine that prevents GBS infection would have 80% efficacy and 90% maternal coverage and would prevent 107,000 neonatal deaths [53].

Despite all of these efforts, the global spread of antibiotic use has led to an increase in bacterial resistance, especially in vulnerable patients, such as pregnant women and neonates, which are complex categories to treat. The increased use of antibiotics treating GBS without adequate bacteriological screening is a factor that raises this concern. Recent studies demonstrated increased resistance to antibiotics, including macrolides and lincosamides, and recently growing resistance to penicillin or fluoroquinolones has been reported [49,54]. A study conducted in China in 2016 demonstrated increased resistance to erythromycin (0–86%), clindamycin (4–84%), and tetracycline (23–96%) [55]. Similarly, a Romanian study showed increased resistance to erythromycin in pregnant patients with GBS infection [56].

### Limitations

Due to limited resources, we admit the study has several limitations. First, the serotype of the identified strains was not identified. Thus, data about GBS serotype distribution in the studied population are insufficient to determine a vaccine prototype. Secondly, the rate of mother-to-fetus transmission was not determined, as it was not a focus point of this survey. Thirdly, this monocentric designated study analyzed the colonization rate and antimicrobial resistance profile in a sample population from a single healthcare facility. Still, the GBS resistance rate can vary by geographic area in Romania. Fourthly, because of the study design, we cannot identify which obstetrical characteristic is significantly associated with GBS colonization.

However, this is the first study in Romania to evaluate maternal GBS colonization and the antibiotic resistance profile in pregnant women. 

## 5. Conclusions

Screening for maternal colonization with GBS remains important in the management of pregnancies worldwide. 

Our study highlights the importance of GBS screening in pregnant patients and antibiotic susceptibility testing for each positive strain. Like the rest of the world, Romania fights to minimize the irrational use of antibiotics caused by empiric treatment.

In our study, GBS colonization had a prevalence of 17.3%. We did not find an association between the antimicrobial susceptibility pattern for the four tested antibiotic classes and gravidity, pregnancy type, history of intrauterine fetal death, or history of antibiotic allergies, but we demonstrated an association between the moment of labor and MICs for clindamycin.

We found that the natural sensitivity of GBS to penicillin is still preserved. However, the resistance of the strains to the second-line therapy, erythromycin and clindamycin, was significantly high (25% and 23.7%). In the battle with antimicrobial resistance in all patients with penicillin allergies, GBS susceptibility testing should be performed before prescribing second-line therapies.

## Figures and Tables

**Table 1:** Demographic characteristics of the studied group according to inclusion criteria.

Age Group (Years)	Number	Percentage (%)
18–19	2	1.3%
20–29	67	44%
30–39	68	44.8%
>40	15	9.9%

**Table 2:** Obstetrical characteristics of the study group.

Variables	Categories	Number	Percentage (%)
Gravidity	Primigravida Multigravida	77 75	50.7% 49.3%
Pregnancy type	Single pregnancy Multiple pregnancies	151 1	99.3% 0.7%
Preterm labor among participants (<37 weeks)	Yes No	9 143	5.9% 94.1%
History of intrauterine fetal death	Yes No	2 150	1.3% 98.7%
History of antibiotic allergies	Yes No	2 150	1.3% 98.7%

**Table 3:** Antimicrobial susceptibility patterns of the tested strains.

Antimicrobial Agent	MIC Breakpoints * (μg/μL)	Number of Strains	Percentage (%)
Penicillin	Susceptible ^a^ (≤0.125)	152	100%
	Susceptible, increased exposure	0	0%
	Resistant (>0.125)	0	0%
Erythromycin	Susceptible (≤0.25)	103	67.8%
	Susceptible, increased exposure	11	7.2%
	Resistant (>0.5)	38	25%
Clindamycin	Susceptible (≤0.5)	115	75.6%
	Susceptible, increased exposure	1	0.7%
	Resistant (>0.5)	36	23.7%
Linezolid	Susceptible (≤2)	149	98%
	Susceptible, increased exposure	0	0%
	Resistant (>2)	3	2%

* MIC breakpoints (μg/μL) according to EUCAST 2020. ^a^ No strains resistant to penicillin were recorded after retesting them.

## Data Availability

Data supporting the reported results are available from the authors.
